# Optimizing sweet fennel growth and quality: the impact of cobalt supplement on vegetative growth, yield, and chemical composition

**DOI:** 10.1186/s12870-025-07314-y

**Published:** 2025-09-16

**Authors:** Nadia Gad, M. E. Fekry Ali, Eman Ali Abd Elrahman, Hanan G. Ismail, Ahmed Fathy Yousef

**Affiliations:** 1 Plant Nutrition Department, National Research Centre Dokki, Cairo, Egypt; 2 Department of Soil and Agricultural Chemistry, Faculty of Agriculture (Saba Basha), Alexandria University, Alexandria, 21531 Egypt; 3 Department of Horticulture, College of Agriculture, University of Al-Azhar (Assiut Branch), Assiut, 71524 Egypt

**Keywords:** Agricultural practices, Essential oils, Macronutrients, Micronutrients, Toxicity threshold, Yield parameters

## Abstract

This study investigated the effects of cobalt treatments on the growth, yield, and chemical composition of sweet fennel (*Foeniculum vulgare* cv. Dulce) in both pot and field experiments conducted over two growing seasons. The experimental design was structured as a completely randomized block design. In the pot experiment, cobalt was applied at concentrations of 0.0, 2, 4, 6, 8, 10, 12, 14, 16, 18, and 20 mg L⁻¹. For the field experiment, the concentrations tested were refined to 0.0, 4, 8, 12, 16, and 20 mg L⁻¹ based on preliminary results to better assess the impact on sweet fennel growth and yield. Vegetative growth, yield parameters, and chemical constituents were evaluated 60 and 120 days after sowing. The results indicated that a cobalt concentration of 16 mg L⁻¹ significantly enhanced plant growth, yield, and nutritional quality. Specifically, plant height, leaf number, fresh and dry weights, bulb dimensions, and total yield per feddan showed substantial increases compared to the control. Chemical analysis revealed improvements in macronutrients, micronutrients, total proteins, carbohydrates, soluble sugars, phenolic content, vitamins C and A, and oil content at this optimal cobalt level. However, higher concentrations (20 mg L⁻¹) led to a slight decline in some metrics, suggesting a potential toxicity threshold. The composition of essential oils also improved with cobalt treatment, with significant increases in key components such as *α*-pinene, camphene, and fenchone. These findings demonstrate that cobalt supplementation at 16 mg L⁻¹ can significantly enhance the growth, yield, and quality of sweet fennel, although excessive levels may have adverse effects.

**Graphical abstract:**

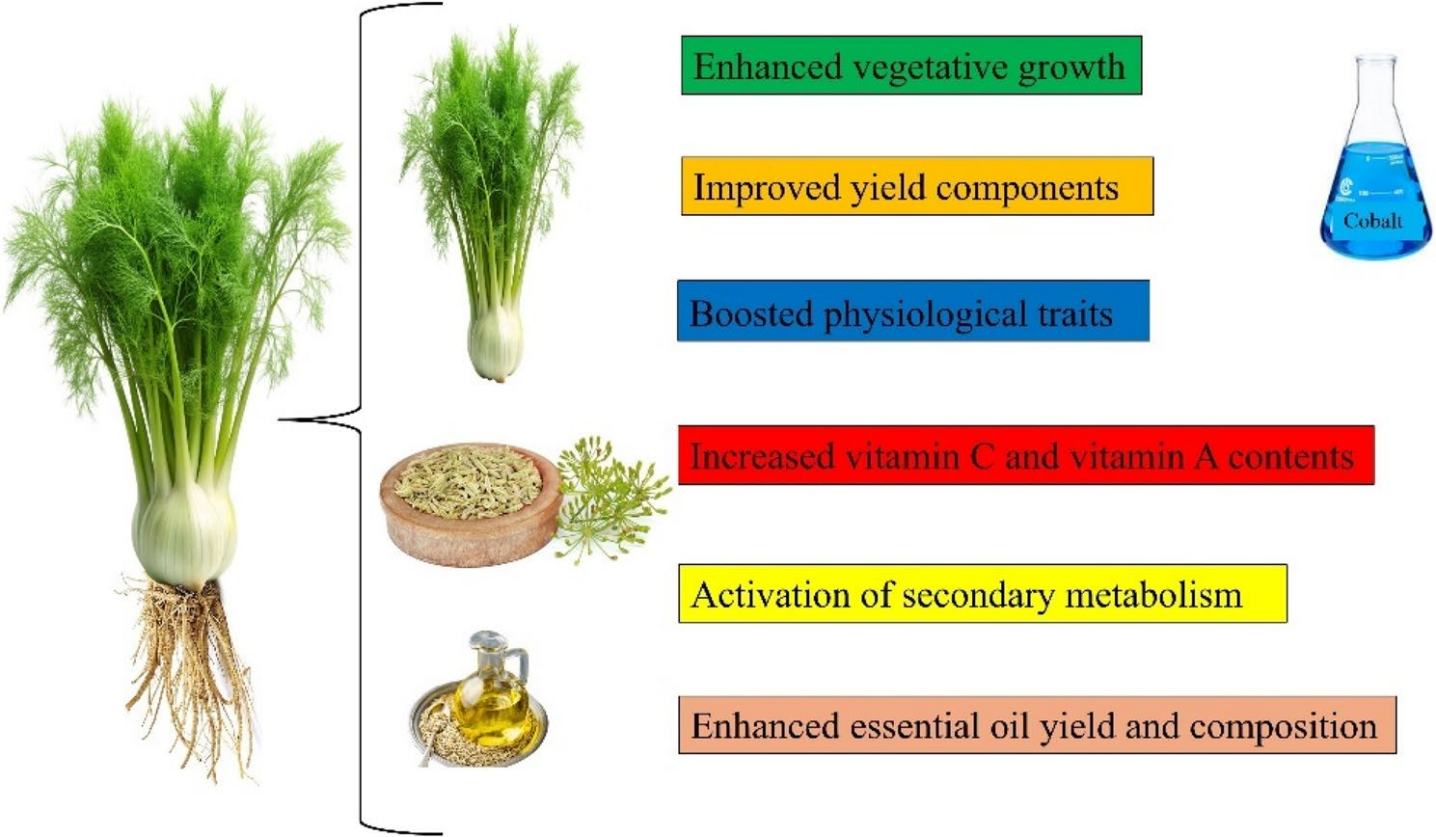

**Supplementary Information:**

The online version contains supplementary material available at 10.1186/s12870-025-07314-y.

## Introduction

Sweet fennel (*Foeniculum vulgare*), a member of the Apiaceae family, is a widely cultivated aromatic and medicinal plant known for its culinary, industrial, and pharmaceutical applications [1]. Its essential oils and bioactive compounds have been extensively studied for their antioxidant, antimicrobial, and anti-inflammatory properties [2]. Globally, sweet fennel is a high-value crop, particularly in Mediterranean countries, India, and Egypt, where it contributes significantly to the spice and essential oil markets [3]. In Egypt alone, fennel cultivation spans thousands of hectares, supporting local economies and export industries due to its demand in flavoring, traditional medicine, and cosmetic products [4, 5]. However, the growth, yield, and quality of sweet fennel are influenced by various environmental and agronomic factors, including nutrient availability and soil conditions [6]. Among these factors, micronutrients play a critical role in plant growth and metabolism, with cobalt (Co) emerging as an essential element that influences physiological processes in plants [7].

Cobalt is a trace element that has been shown to enhance plant growth, nitrogen fixation, and stress tolerance in certain crops [8]. It is a component of vitamin B12 and is involved in enzymatic activities that regulate plant metabolism [9]. For example, in legumes such as faba beans (*Vicia faba* L.) and black gram (*Vigna mungo* L.), cobalt improves nitrogen fixation by supporting rhizobial activity, leading to increased biomass and yield [10, 11]. Similarly, in cereals like wheat (*Triticum aestivum* L.), cobalt mitigates abiotic stress and enhances nutrient uptake [12]. In non-leguminous crops such as tomatoes (*Solanum lycopersicum* L.) and onions (*Allium cepa* L.), optimal cobalt levels have been linked to improved growth, yield, and nutritional quality [13, 14]. However, the role of cobalt in plant physiology remains understudied, particularly in aromatic and medicinal plants like sweet fennel While cobalt deficiency can limit plant growth, excessive concentrations may lead to toxicity, highlighting the need for optimal application rates to maximize crop productivity and quality [10, 15]. Recent studies have demonstrated the potential of cobalt to improve growth parameters, yield, and secondary metabolite production in various crops [12, 13]. However, the specific effects of cobalt on sweet fennel, particularly its impact on growth, yield components, and quality, have not been thoroughly investigated. Understanding the optimal cobalt concentrations for sweet fennel cultivation could provide valuable insights for improving its agronomic practices and enhancing its economic value.

In this context, the present study was conducted to evaluate the effects of cobalt on the growth, yield, and quality of sweet fennel. The study hypothesized that optimal concentrations of cobalt would significantly enhance the growth, yield, and quality parameters of sweet fennel. The study aimed to (1) identify the optimal cobalt concentrations for sweet fennel growth and yield, (2) assess the impact of cobalt on yield components and quality parameters, and (3) provide practical recommendations for cobalt application in sweet fennel cultivation.

## Materials and methods

### Soil analysis

Soil samples were collected from the upper 0–30 cm layer, air-dried, crushed, and passed through a 2 mm sieve [16]. Soil texture was determined using the pipette method as described by Page [17], while calcium carbonate (CaCO₃) content was assessed using the calcimeter method outlined by Jackson (18). Organic matter content was analyzed through dichromate oxidation. Soil salinity was determined by measuring the electrical conductivity (EC) of a 1:1 soil-water extract using an EC meter (LOvibond 200 con, Germany), and soil pH was measured in a 1:2.5 soil-water suspension with a pH meter (Hanna Instruments pH 211, Romania) [18]. Available nitrogen (N) was extracted using 1% K₂SO₄ at a 1:5 soil-to-solution ratio, followed by distillation of 20 mL of the extract with 1 g of Devarda’s alloy in a micro-Kjeldahl apparatus [18]. For available phosphorus (P), extraction was performed using 0.5 M sodium bicarbonate (pH 8.5), and quantification was carried out using the stannous chloride method, with absorbance readings taken at 660 nm using a spectrophotometer (Unico 2000UV, Germany) [18]. Potassium (K) levels were measured using a flame photometer (Jenway 7PFP, England) [18]. Micronutrients (Fe, Mn, Zn, Cu) were analyzed according to Page [17], and cobalt concentrations were determined following the method described by Ryan, Estefan [19]. Key soil characteristics are presented in Table 1.

**Table 1:** Some physical and chemical properties of soil

**Physical properties**							
**Particle size distribution %**				**Soil moisture constant %**			
**Sand** **7**0**.8**	**Silt** 25.6	**Clay** 3.6	**Soil texture** **Sandy loam**	**Saturation** 32.0	**FC** 19.2	**WP** 6.1	**AW** 13.1

**Chemical properties**											
				**Soluble cations (mmolcL**^**-1**^**)**				**Soluble anions (mmolcL**^**-1**^**)**			
pH1:2.5 8.49	EC(dS m^-1^)1.74	CaCO_3_% 3.4	OM%0.20	Ca^++^ 0.8	Mg^++^ 0.5	K^+^ 1.6	Na^+^1.80	HCO_3_^-^0.3	CO_3_ 0.0	Cl^-^ 1.9	SO_4_^--^0.5

Cobalt			Available macronutrients			Available micronutrients			
ppm			mg 100 g^-1^ soil			ppm			
Soluble 0.35	Available 4.88	Total 9.88	N 15.1	P 13.3	K 4.49	Fe 4.46	Mn 2.71	Zn 4.52	Cu 5.2

Where: *FC *Field Capacity (%), *WP *Wilting Point (%), *AW *Available Water (%)

### Experimental site and design

This stepwise approach was based on both preliminary results and existing literature, which indicate that cobalt application in the range of 5–20 mg L⁻¹ has shown physiological benefits across various plant species, including legumes, cereals, and aromatic crops. Specifically, prior studies have reported that foliar or soil applications of cobalt within this range enhance nitrogen metabolism, stress tolerance, and the biosynthesis of secondary metabolites such as essential oils [8, 11, 14, 15]. The selected concentrations allowed us to capture the full response curve, including the point of optimal efficacy (16 mg L⁻¹) and potential toxicity threshold (20 mg L⁻¹), ensuring a comprehensive assessment of physiological relevance for this species.

#### Preliminary pots experiment

A preliminary pot experiment was conducted in the wirehouse of the National Research Centre, located on El-Bohooth Street, Dokki, Cairo, Egypt. Plastic pots with a capacity of 10 kg were filled with clay soil collected from a farm in Nobaria, Beheira Governorate, Egypt. Average day and night temperatures were approximately 35 ± 2 °C and 24 ± 2 °C, respectively, with relative humidity around 50 ± 5%. Sweet fennel seeds (*Foeniculum vulgare* cv. Dulce) were sown in August 2021 in the nursery using the same soil type (sandy loam) intended for the subsequent field trials. The pot experiment included 11 cobalt treatments (0.0, 2, 4, 6, 8, 10, 12, 14, 16, 18, and 20 mg L⁻¹), each with 3 replicates. Five plants per replicate were randomly sampled for measurements, totaling 15 plants per treatment. Once the seedlings reached the third true leaf stage, they were irrigated once with 500 mL per pot of cobalt sulfate solutions at concentrations of 0.0, 2, 4, 6, 8, 10, 12, 14, 16, 18, and 20 mg L⁻¹, applied as a soil drench. Subsequent irrigation was performed using tap water to maintain soil moisture near field capacity.

#### Field experiments

Field experiments were carried out at the Research and Production Station of the National Research Centre in El-Nubaryia, Beheira Governorate, Delta Egypt (30° 23’ 48” N, 30° 18’ 59” E) during the 2022/2023 and 2023/2024 growing seasons. Climatic conditions during the 2022/2023 and 2023/2024 growing seasons (August–January) included hot, dry summers and cool, moderately wet winters. Bi-weekly averages of temperature, relative humidity, and rainfall are provided in Table S1 and Table S2. aThe experimental design followed a completely randomized block design with three replicates. Each plot measured 15 m² (3 m × 5 m) and consisted of four rows, with ten plants per row [Each replicate plot contained 40 plants (4 rows × 10 plants), totaling 120 plants per treatment]. Sweet fennel seeds were sown on 20th and 22nd August during both seasons. Once the seedlings reached the third true leaf stage, they were irrigated once with cobalt concentrations. Based on preliminary results, the cobalt concentrations tested were 0.0, 4, 8, 12, 16, and 20 mg L⁻¹, which were found to influence sweet fennel growth and yield responses.

All necessary agricultural practices, including fertilization and irrigation, were conducted in accordance with the guidelines provided by the Ministry of Agriculture, Egypt. A fertilizer injector was used to mix fertilizers with irrigation water, ensuring efficient delivery through the irrigation system. Fertilization was administered in three stages: the first dose after transplanting, the second one month later, and the third during the flowering stage. Prior to planting, the soil was sterilized using 100 kg fadan^−1^ (4200 m²) of agricultural sulfur, produced by Abu Qir Fertilizer and Chemical Industries. A basal dose of 60 kg Fadan⁻¹ P₂O₅ (15.5% CaH₆O₉P₂) and 20 m³ of decomposed organic fertilizer were incorporated into the soil. Additionally, 120 kg N Fadan⁻¹ (33.5% NH₄NO₃) and 50 kg Fadan⁻¹ K₂O (50% potassium sulfate) were applied in two equal splits at 30 and 60 days after transplanting, following the recommendations of the Ministry of Agriculture, Egypt [20].

### Measurements of vegetative growth

Sixty days after sowing, various vegetative growth parameters of sweet fennel plants were measured to evaluate plant development and biomass accumulation. A random sample of five plants from each row was selected for analysis. Plant height was measured from the base of the stem at soil level to the apex of the main shoot using a measuring tape, recorded in centimeters (cm). The number of leaves per plant was counted, including only fully expanded leaves. The fresh weight of the whole plant, as well as its leaves and bulb, was measured immediately after harvesting using a digital balance, recorded in grams (g). To determine dry weights, the plant parts (leaves and bulb) were separated and oven-dried at 70 °C for 48 h or until a constant weight was achieved. The dry weights were then measured and recorded in grams (g).

### Measurements of yield characteristics

At harvest time, 120 days after sowing, yield parameters of sweet fennel were measured to assess crop productivity and the impact of experimental treatments. These parameters included: (1) Bulb Height: The vertical length of the bulb was measured from the base (where it connects to the roots) to the apex (top of the bulb) using a measuring tape, recorded in centimeters (cm). (2) Bulb Diameter: The horizontal width of the bulb was measured at its widest point using a caliper or measuring tape, recorded in centimeters (cm). (3) Bulb Weight: The fresh weight of individual bulbs was measured immediately after harvesting using a digital balance, recorded in grams (g). (4) Total Yield: The total yield of sweet fennel bulbs was calculated per unit area and expressed in tons per feddan (ton fed^−1^).

### Determination of chemical constituents

At harvest time, 120 days after sowing, a random sample of 20 bulbs from each plot was collected to determine all chemical constituents. Fresh sweet fennel bulbs were carefully selected, washed, and homogenized to create a uniform sample for analysis. These samples were analyzed to evaluate the biochemical composition of sweet fennel bulbs according to the method for each component separately, as follows:

#### Minerals compositions analysis

The study involved the quantification of essential elements, including nitrogen (N), phosphorus (P), potassium (K), manganese (Mn), zinc (Zn), copper (Cu), iron (Fe), and cobalt (Co), in sweet fennel bulbs using high-performance liquid chromatography (HPLC) [21]. Samples of sweet fennel bulbs were homogenized and underwent acid digestion to extract the target elements. The digested samples were filtered and appropriately diluted for analysis via HPLC. A specific mobile phase and column were employed to separate the elements, and detection was carried out using a UV-Vis detector. Calibration curves were generated using standard solutions for each element, and the concentrations of N, P, K, Mn, Zn, Cu, Fe, and Co were determined based on the corresponding peak areas. The results were reported in milligrams per 100 g of fresh weight (mg 100 g^−1^ FW).

#### Total protein

The total protein content in sweet fennel bulbs was quantified using the Kjeldahl method [22], a well-established and reliable technique for protein analysis. A random sample of 20 bulbs from each plot was homogenized, and a representative portion (approximately 0.5 g) was precisely weighed and placed into a digestion flask. The sample was digested using concentrated sulfuric acid (H₂SO₄) in the presence of a catalyst (potassium sulfate, K₂SO₄) to convert organic nitrogen into ammonium sulfate. Digestion was performed at a high temperature (~ 400 °C) until the solution turned clear, indicating complete digestion. The digested sample was then transferred to a distillation apparatus. Sodium hydroxide (NaOH) was added to the digested solution to release ammonia (NH₃) gas, which was distilled and collected in a receiving flask containing a known volume of boric acid (H₃BO₃) solution. The ammonia absorbed in the boric acid solution was titrated with a standardized hydrochloric acid (HCl) solution (0.1 N), using methyl red as an indicator. The titration endpoint was marked by a color change from green to pink. The total nitrogen content was calculated based on the volume of HCl consumed during titration and the normality of the HCl solution. The protein content was derived by multiplying the total nitrogen content by a conversion factor of 6.25, which is standard for plant materials. The total protein percentage was calculated using the following formula:

$$\begin{aligned} &\mathrm{Total}\;\mathrm{Protein}\;(\%)\;\;\\&=\;\frac{\text{V}\times\:\text{Normality}\:\text{of}\:\text{HCl}\times\:1.401\times\:6.25}{\text{W}} \end{aligned}$$

where: V is volume of HCl (mL), 1.401 is the milliequivalent weight of nitrogen; 6.25 is the conversion factor for converting nitrogen to protein, Wis weight of Sample (g).

#### Determination of total carbohydrates

The total carbohydrates content in sweet fennel bulbs was determined using the phenol-sulfuric acid method [23], a widely used colorimetric technique for carbohydrate quantification. A representative portion of the homogenate was weighed and subjected to extraction using distilled water. The extracted solution was then mixed with 5% phenol solution, followed by the addition of concentrated sulfuric acid (H₂SO₄). The reaction between carbohydrates, phenol, and sulfuric acid produced a yellow-orange color, the intensity of which was proportional to the carbohydrate concentration. The absorbance of the solution was measured at 490 nm using a UV-Vis spectrophotometer (Model: Unico 2000UV, Germany). A standard curve was prepared using glucose as a reference, with known concentrations of glucose solutions treated in the same manner as the samples. The total carbohydrates content in the sweet fennel bulbs was calculated based on the standard curve and expressed as grams per 100 g of fresh weight (g 100 g^−1^ FW).

#### Determination of total soluble sugars

The total soluble sugars content in sweet fennel bulbs was determined using the anthrone-sulfuric acid method [24], a widely recognized colorimetric technique for quantifying soluble sugars. A representative portion of the homogenate was weighed and extracted using 80% ethanol to isolate the soluble sugars. The extract was centrifuged to remove insoluble residues, and the supernatant was collected for analysis. The supernatant was mixed with anthrone reagent (prepared by dissolving anthrone in concentrated sulfuric acid), which reacts with soluble sugars to produce a blue-green color. The intensity of the color is proportional to the concentration of soluble sugars in the sample. The mixture was heated in a water bath at 100 °C for 10 min to complete the reaction, then cooled to room temperature. The absorbance of the solution was measured at 620 nm using a UV-Vis spectrophotometer (Model: Unico 2000UV, Germany). A standard curve was prepared using glucose as a reference, with known concentrations of glucose solutions treated in the same manner as the samples. The total soluble sugars content in the sweet fennel bulbs was calculated based on the standard curve and expressed as milligrams per gram of fresh weight (mg g^−1^ FW).

#### Determination of total soluble solids

To determine the total soluble solids (T.S.S.) content in sweet fennel bulbs, the bulbs were meticulously washed, processed, and homogenized to produce a smooth puree. The supernatant was separated from any sediment, and the resulting juice was filtered as needed. The T.S.S. content was measured using a digital refractometer (Model: Milwaukee MA873, Milwaukee Co., United States), with results reported in Brix (°Bx) units. The procedure followed established standard protocols [25].

#### Determination of phenol content

The total phenol content in sweet fennel bulbs was determined using the Folin-Ciocalteu method [26], a widely used colorimetric assay for quantifying phenolic compounds. A representative portion of the homogenate was weighed and extracted using 80% methanol or another suitable solvent to isolate phenolic compounds. The extract was centrifuged to remove insoluble residues, and the supernatant was collected for analysis. The supernatant was mixed with Folin-Ciocalteu reagent, which reacts with phenolic compounds to produce a blue color. After thorough mixing, sodium carbonate (Na₂CO₃) solution was added to the mixture to stabilize the reaction. The solution was incubated in the dark at room temperature for 30 min to allow the color to develop fully. The absorbance of the solution was measured at 765 nm using a UV-Vis spectrophotometer (Model: Unico 2000UV, Germany). A standard curve was prepared using gallic acid as a reference, with known concentrations of gallic acid solutions treated in the same manner as the samples. The total phenol content in the sweet fennel bulbs was calculated based on the standard curve and expressed as milligrams of gallic acid equivalents per gram of fresh weight (mg GAE g^−1^ FW).

#### Determination of vitamin C (as L-Ascorbic Acid)

The vitamin “C” content in sweet fennel bulbs was determined using High-Performance Liquid Chromatography (HPLC) according to standard protocols (HPLC-UV or HPLC-DAD) [25]. A representative portion of the sample was weighed, and vitamin “C” was extracted using a suitable solvent such as 3% metaphosphoric acid (HPO₃) to prevent the oxidation of ascorbic acid during extraction. The extract was filtered to remove solid impurities, and the filtrate was collected for analysis. The sample was analyzed using an HPLC device equipped with a UV detector or Diode Array Detector (DAD). A C18 column was used for compound separation, and a suitable mobile phase was selected, such as a mixture of acidified water (with 0.1% formic acid or phosphoric acid) and methanol or acetonitrile in specific ratios. The use of acidified water helps stabilize ascorbic acid and improve peak shape, while the choice between methanol and acetonitrile as the organic modifier was based on optimizing retention time, resolution, and sensitivity under the chromatographic conditions used. These combinations were selected based on method validation to ensure reproducibility, efficient elution, and compatibility with UV detection. The detection wavelength was set to 245 nm, where ascorbic acid absorbs optimally. A calibration curve was prepared using known concentrations of pure ascorbic acid as a reference. The concentration of vitamin “C” in the samples was calculated based on the peak areas measured by the device, and the results were expressed as milligrams of ascorbic acid per 100 g of fresh weight (mg 100 g^−1^ FW).

#### Determination of vitamin A (as Beta-Carotene)

The Vitamin A content in sweet fennel bulbs, expressed as beta-carotene, was determined using High-Performance Liquid Chromatography (HPLC) [27]. A representative portion of the homogenate was weighed and extracted using acetone to isolate beta-carotene. The extract was centrifuged to remove insoluble residues, and the supernatant was filtered through a 0.45 μm membrane filter to ensure clarity before injection into the HPLC system. The analysis was performed using a C18 reverse-phase column and a UV-Vis or diode array detector (DAD). The mobile phase consisted of a gradient mixture of acetonitrile and methanol (70:30 v/v) at a flow rate of 1.0 mL min^−1^. The detection wavelength was set to 450 nm, which is optimal for beta-carotene absorption. A calibration curve was prepared using known concentrations of pure beta-carotene standard. The concentration of beta-carotene in the sweet fennel bulb samples was calculated based on the peak areas obtained from the HPLC chromatogram. The results were expressed as grams of beta-carotene per 100 g of fresh weight (g 100 g^−1^ FW).

#### Determination of essential oil content

The essential oil content in sweet fennel bulbs was determined using the hydrodistillation method (Clevenger apparatus) [28]. This method is widely used for extracting and quantifying essential oils from plant materials. A representative sample of the chopped bulbs (approximately 100 g) was weighed and placed in a round-bottom flask along with distilled water. The flask was connected to a Clevenger apparatus, and the mixture was heated to boiling. The oil was separated from the water in the graduated tube of the Clevenger apparatus due to its lower density. The volume of the extracted oil was measured directly from the graduated tube, and the oil content was calculated using the following formula:

$$\:\mathrm{Essential}\;\mathrm{Oil}\;\mathrm{Content}\;\;\left(\mathrm{mL}\;\mathrm{kg}^{-1}\right)\frac{Volume\:of\:Oil\:\left(mL\right)}{Weight\:of\:Sample\:\left(kg\right)}$$

#### Determination the percentage composition of the essential oils

The percentage composition of the essential oils in oil content of sweet fennel bulbs was determined using Gas Chromatography-Mass Spectrometry (GC-MS) analysis (model 7890B, Agilent Co. China) at central laboratories Network, National Research Centre, Cairo, Egypt. The extracted oil was injected into the GC-MS system, where individual components were separated using a capillary column and identified based on their mass spectra. The analysis was performed in triplicate to ensure accuracy, and compounds present in trace amounts (< 0.1%) were marked as “T.” The percentage composition of each compound was calculated using the formula:

$$\begin{aligned} &\mathrm{Percentage}\;\mathrm{Composition}\;\left(\%\right)\;\\&=\:\frac{Peak\:Area\:of\:the\:Compound}{Total\:Peak\:Area\:of\:All\:Compounds}\times\:100 \end{aligned}$$

Compounds were identified by comparing retention times and mass spectra with known standards or databases (e.g., NIST library).

###  Statistical analysis

All data were subjected to statistical analysis using the SAS software package (SAS, 1996). Treatment means were compared using the Least Significant Difference (LSD) test, as outlined by Snedecor and Cochram [29], to identify significant differences at a 5% probability level (*p* ≤ 0.05).

## Results

### Vegetative growth of pot experiment plants

Table 2 illustrates the impact of various cobalt treatments on the vegetative growth of sweet fennel plants 60 days after sowing in pots experiment. The most significant growth was observed at a cobalt concentration of 16 mg L^−1^. Specifically, the plant height reached 76.4 cm, marking an increase of approximately 54.03% compared to the control. The number of leaves peaked at 12.12, showing a 100.66% rise relative to the control. In terms of fresh weight, the leaves weighed 101.2 g, a 41.94% increase, while the bulb reached 68.81 g, up by 63.13%. The total fresh weight per plant was 170.01 g, reflecting a 49.82% increase. For dry weight, the leaves weighed 63.08 g (a 54.72% increase), and the bulb weighed 39.20 g (a 166.30% increase). The total dry weight per plant was 102.88 g, an 85.40% increase compared to the control.

**Table 2:** Vegetative growth of sweet fennel plant which sowing in pots experiments as affected by cobalt addition after 60 days from sowing (mean of two seasons)

Cobalt Treatments (mg L^−1^)	Plant height (cm)	Number of leaves	Fresh weight/plant (g)			Dry weight/plant (g)		
			Leaves	Blub	Total	Leaves	bulb	Total
Control	49.60 ± 1.21 g	6.04 ± 0.07 g	71.30 ± 0.90 g	42.18 ± 0.63 g	113.48 ± 4.35 g	40.77 ± 0.50 g	14.72 ± 0.73 g	55.49 ± 0.85 g
2	51.80 ± 0.55f	6.20f ± 0.08 g	73.61 ± 0.48f	44.34 ± 0.65f	117.95 ± 0.99f	42.62 ± 0.61f	18.24 ± 0.30f	60.86 ± 0.60f
4	56.40 ± 0.88e	7.12 ± 0.06e	79.08 ± 1.38e	48.60 ± 0.76e	127.68 ± 1.42e	45.19 ± 1.18e	20.90 ± 0.46e	66.09 ± 0.84e
6	58.10 ± 1.37d	7.39 ± 0.11d	82.11 ± 0.94d	51.19 ± 0.63d	133.30 ± 1.56d	46.22 ± 0.11d	22.18 ± 0.20d	68.32 ± 0.72d
8	65.70 ± 1.46c	7.81 ± 0.16c	86.00 ± 1.04c	54.40 ± 0.76c	140.40 ± 0.81c	49.42 ± 0.31c	25.60 ± 0.28c	75.02 ± 0.17c
10	65.40 ± 0.86c	8.06 ± 0.16b	89.21 ± 1.12b	57.01 ± 1.13b	146.22 ± 1.75b	51.70 ± 0.65b	27.00 ± 0.96b	78.70 ± 0.53b
12	68.00 ± 1.59b	9.21 ± 0.08a	94.13 ± 1.64a	62.31 ± 0.41a	156.44 ± 2.05a	55.60 ± 0.28a	32.31 ± 1.11a	87.91 ± 1.16a
14	71.50 ± 0.85a	10.56 ± 0.10a	97.04 ± 1.16a	65.51 ± 0.84a	162.55 ± 0.55a	58.80 ± 0.43a	35.50 ± 0.26a	94.30 ± 0.97a
16	76.40 ± 1.69a	12.12 ± 0.18a	101.20 ± 0.37a	68.81 ± 0.61a	170.01 ± 2.05a	63.08 ± 0.34a	39.20 ± 0.54a	102.88 ± 1.94a
18	73.30 ± 1.32a	10.6 ± 0.13a	98.11 ± 0.70a	66.71 ± 0.93a	164.42 ± 1.66a	61.80 ± 0.61a	37.00 ± 0.62a	98.80 ± 0.89a
20	69.80 ± 1.46b	10.80 ± 0.05a	94.61 ± 1.03a	63.23 ± 0.34a	157.84 ± 1.39a	59.40 ± 0.33a	35.10 ± 0.19a	94.50 ± 0.62a
LSD %5	2.1	0.27	2.4	2.2	4.36	2.05	2.51	2.21

Values within a column (Mean ± SD) followed by different lowercase letters are significantly different according to LSD test at *p* ≤ 0.05

### Field experiments

#### Vegetative growth

Table 3 details the impact of various cobalt treatments on the vegetative growth of sweet fennel plants 60 days after sowing in an open farm setting. The most significant growth was observed at a cobalt concentration of 16 mg L^−1^. Specifically, the plant height reached 80.6 cm, representing an increase of approximately 44.70% compared to the control. The number of leaves peaked at 14.82, showing a 107.56% rise relative to the control. In terms of fresh weight, the leaves weighed 104.31 g, a 36.35% increase, while the bulb reached 75.44 g, up by 56.51%. The total fresh weight per plant was 179.7 g, reflecting a 46.45% increase. For dry weight, the leaves weighed 71.32 g (a 59.80% increase), and the bulb weighed 41.25 g (a 142.16% increase). The total dry weight per plant was 112.57 g, an 81.74% increase compared to the control. These findings demonstrate that a cobalt treatment of 16 mg L^−1^ significantly enhances the vegetative growth of sweet fennel plants across various parameters. However, it is noteworthy that at higher concentrations (20 mg L^−1^), some growth metrics showed a slight decline, indicating a potential toxicity threshold.

**Table 3:** Vegetative growth of sweet fennel plant which planted in field experiments as affected by Cobalt addition after 60 days from sowing (mean of two seasons)

Cobalt Treatments (mg L^−1^)	Plant height (cm)	Number of leaves	Fresh weight/plant (g)			Dry weight/plant (g)		
			Leaves	Blub	Total	Leaves	bulb	Total
Control	55.70 ± 1.82d	7.14 ± 0.51d	76.50 ± 2.11d	48.20 ± 1.62d	122.70 ± 2.89d	44.63 ± 2.31d	17.03 ± 0.94d	61.94 ± 2.42d
4	60.80 ± 1.43c	8.66 ± 0.63c	82.19 ± 1.87c	51.70 ± 1.32c	133.90 ± 2.45c	48.14 ± 1.97c	19.32 ± 0.87c	67.16 ± 2.11c
8	66.90 ± 1.67b	10.86 ± 0.72b	88.20 ± 2.05b	59.51 ± 1.54b	147.70 ± 2.67b	56.08 ± 2.15b	26.01 ± 0.99b	82.09 ± 2.33b
12	73.80 ± 1.92a	12.73 ± 0.85a	95.41 ± 2.36a	66.90 ± 1.77a	162.30 ± 3.07a	62.71 ± 2.47a	33.13 ± 1.14a	95.84 ± 2.85a
16	80.60 ± 2.15a	14.82 ± 0.95a	104.31 ± 2.64a	75.44 ± 1.98a	179.70 ± 3.44a	71.32 ± 2.76a	41.25 ± 1.28a	112.57 ± 3.19a
20	75.90 ± 1.78a	12.36 ± 0.88a	98.62 ± 2.42a	70.02 ± 1.82a	168.60 ± 3.18a	66.43 ± 2.55a	35.73 ± 1.18a	102.16 ± 2.98a
LSD %5	4.59	2.09	5.01	3.49	6.1	5.48	2.23	5.17

Values within a column (Mean ± SD) followed by different lowercase letters are significantly different according to LSD test at *p* ≤ 0.05

#### Yield characteristics

Table 4 details the impact of various cobalt treatments on the yield parameters of sweet fennel plants 120 days after sowing in field experiments. The most significant improvements were observed at a cobalt concentration of 16 mg L^−1^. Specifically, the bulb height reached 11.58 cm, representing an increase of approximately 18.4% compared to the control. The bulb diameter peaked at 13.82 cm, showing a 15.8% rise relative to the control. In terms of bulb weight, the highest value was 77.5 g, a 46.5% increase compared to the control. The total yield per feddan reached 15.80 tons Fed^−1^, reflecting a 20.2% increase. These findings demonstrate that a cobalt treatment of 16 mg L^−1^ significantly enhances the yield parameters of sweet fennel plants across various metrics. However, it is noteworthy that at higher concentrations (20 mg L^−1^), some yield metrics showed a slight decline, indicating a potential toxicity threshold. For example, the bulb height decreased to 10.78 cm, and the total yield dropped to 14.95 tons per feddan, suggesting that excessive cobalt levels may adversely affect plant growth and productivity.

**Table 4:** Yield parameters of sweet fennel plant which planted in field experiments as affected by cobalt addition after 120 days from sowing (mean of two seasons)

Cobalt Treatments (mg L^−1^)	Bulb Height (cm)	Bulb diameter (cm)	Bulb Weight (g)	Total Yield Ton Fed^−1^
Control	9.78 ± 0.12d	11.93 ± 0.25d	52.90 ± 2.25d	13.15 ± 0.41d
4	10.66 ± 0.10c	12.37 ± 0.22c	57.60 ± 1.98c	13.87 ± 0.36c
8	10.91 ± 0.11bc	12.89 ± 0.20b	61.80 ± 2.10b	14.63 ± 0.38b
12	11.26 ± 0.13ab	13.20 ± 0.18a	69.60 ± 2.45a	15.15 ± 0.42a
16	11.58 ± 0.15a	13.82 ± 0.15a	77.50 ± 2.80a	15.80 ± 0.45a
20	10.78 ± 0.14c	13.49 ± 0.16a	73.60 ± 2.60a	14.95 ± 0.43a
LSD %5	0.23	0.42	3.92	0.69

Values within a column (Mean ± SD) followed by different lowercase letters are significantly different according to LSD test at *p* ≤ 0.05

#### Chemical constituents

##### Minerals composition analysis

Table 5 details the impact of various cobalt treatments on the minerals composition analysis of fennel bulbs 120 days after sowing in field experiments. The most significant improvements in macronutrient and micronutrient content were observed at a cobalt concentration of 16 mg L^−1^. For macronutrients, the highest nitrogen (N) content was 1.419%, representing an increase of approximately 62.4% compared to the control (0.874%). Phosphorus (P) content peaked at 0.619%, showing a 40.0% rise relative to the control (0.442%). Potassium (K) content reached its maximum at 1.526%, a 64.2% increase compared to the control (0.929%). In terms of micronutrients, manganese (Mn) content was highest at 58.9 mg L^−1^, an increase of 23.2% compared to the control (47.8 mg kg^−1^). Zinc (Zn) content peaked at 42.0 mg kg^−1^, reflecting a 28.8% rise relative to the control (32.6 mg kg^−1^). Copper (Cu) content reached its maximum at 36.8 mg L^−1^, a 33.3% increase compared to the control (27.6 mg kg^−1^). Iron (Fe) content, however, showed a different trend, with the lowest value of 156 mg kg^−1^ at 20 mg L^−1^ cobalt, representing a 13.8% decrease compared to the control (181 mg kg^−1^). Cobalt accumulation in fennel bulbs exhibited a dose-dependent response, with concentrations rising significantly (*p* ≤ 0.05) from 0.96 mg kg⁻¹ in control plants to 11.71 mg kg⁻¹ at the highest treatment level (20 mg L⁻¹). At the optimal concentration of 16 mg L⁻¹, cobalt content reached 8.06 mg kg⁻¹ (Table 5), reflecting a near-linear relationship between application rates and plant uptake.These findings demonstrate that a cobalt treatment of 16 mg L^−1^ significantly enhances the nutritional status of fennel bulbs across various metrics. However, it is noteworthy that at higher concentrations (20 mg L^−1^), some nutrient metrics showed a slight decline, indicating a potential toxicity threshold. For example, nitrogen content decreased to 1.405%, and iron content dropped to 156 mg L^−1^, suggesting that excessive cobalt levels may adversely affect nutrient uptake and plant health.

**Table 5:** Minerals composition status of fennel bulb which planted in field experiments as affected by cobalt addition after120 days from sowing (mean of two seasons)

Cobalt Treatments (mg L^−1^)	Macronutrients (%)			Micronutrients (mg kg^−1^)				
	*N*	*P*	K	Mn	Zn	Cu	Fe	Cobalt
Control	0.874 ± 0.028e	0.442 ± 0.008e	0.929 ± 0.075e	47.80 ± 1.15e	32.60 ± 0.58e	27.60 ± 0.72e	181.00 ± 2.45a	0.96 ± 0.12f
4	1.253 ± 0.025d	0.468 ± 0.007d	1.163 ± 0.070d	50.60 ± 1.08d	34.00 ± 0.55d	29.20 ± 0.68d	178.00 ± 2.35a	1.18 ± 0.15e
8	1.319 ± 0.023c	0.511 ± 0.006c	1.281 ± 0.065c	53.40 ± 1.02c	36.70 ± 0.52c	32.00 ± 0.65c	172.00 ± 2.25b	3.24 ± 0.18d
12	1.382 ± 0.021b	0.570 ± 0.005b	1.439 ± 0.060b	55.60 ± 0.95b	39.60 ± 0.48b	34.40 ± 0.60b	167.00 ± 2.15c	6.87 ± 0.25c
16	1.419 ± 0.020a	0.619 ± 0.004a	1.526 ± 0.055a	58.90 ± 0.90a	42.00 ± 0.45a	36.80 ± 0.55a	160.00 ± 2.05d	8.06 ± 0.30b
20	1.405 ± 0.022a	0.671 ± 0.003a	1.478 ± 0.058ab	55.90 ± 0.92a	39.70 ± 0.47a	35.00 ± 0.57a	156.00 ± 2.10d	11.71 ± 0.35a
LSD %5	0.064	0.024	0.219	2.4	1.2	1.5	4.91	2.3

Values within a column (Mean ± SD) followed by different lowercase letters are significantly different according to LSD test at *p* ≤ 0.05

##### Total protein, Total carbohydrates, Total soluble sugars, Total Soluble Solids, Phenol Content, Vitamin C, Vitamin A, Oil Content

Table 6 details the impact of various cobalt treatments on the chemical constituents of fennel bulbs 120 days after sowing in field experiments. The most significant improvements in chemical constituents were observed at a cobalt concentration of 16 mg L^−1^. For total proteins, the highest content was 8.86%, representing an increase of approximately 62.3% compared to the control (5.46%). Total carbohydrates peaked at 17.71%, showing a 25.5% rise relative to the control (14.11%). Total soluble sugars reached their maximum at 4.56%, a 41.2% increase compared to the control (3.23%). Total soluble solids content was highest at 8.96%, reflecting a 24.8% increase compared to the control (7.18%). In terms of phenolic content, the highest value was 9.26%, an increase of 34.6% compared to the control (6.88%). Vitamin C content peaked at 14.66 mg 100 g^−1^ FW, showing an 18.5% rise relative to the control (12.37 mg 100 g^−1^ FW). Vitamin A content reached its maximum at 13.56 g 100 g^−1^ FW, a 19.9% increase compared to the control (11.31 g 100 g^−1^ FW). Oil content was highest at 0.0989 mL kg^−1^, reflecting a 25.8% increase compared to the control (0.0786 mL kg^−1^). These findings demonstrate that a cobalt treatment of 16 mg L^−1^ significantly enhances the chemical constituents of fennel bulbs across various metrics. However, it is noteworthy that at higher concentrations (20 mg L^−1^), some chemical metrics showed a slight decline, indicating a potential toxicity threshold. For example, total proteins decreased to 8.78%, total carbohydrates dropped to 17.22%, and total soluble solids content decreased to 8.13%, suggesting that excessive cobalt levels may adversely affect the chemical composition and quality of fennel bulbs.

**Table 6:** Chemical constituents of fennel bulb which planted in field experiments as affected by cobalt addition after 120 days from sowing (mean of two seasons)

Cobalt Treatments (mg L^−1^)	Total Proteins (%)	Total Carbo- Hydrates (%)	Total Soluble Sugars (%)	Total soluble solids (%)	Phenols (%)	Vitamin (C) (mg 100 g^−1^ FW)	Vitamin (A) (g 100 g^−1^ FW)	Essential oil (mL kg^−1^)
Control	5.46 ± 0.58d	14.11 ± 0.30d	3.23 ± 0.04d	7.18 ± 0.27d	6.88 ± 0.31d	12.37 ± 0.15d	11.31 ± 0.18d	0.0786 ± 0.0041d
4	7.83 ± 0.49c	15.32 ± 0.25c	3.32 ± 0.03c	7.76 ± 0.23c	7.53 ± 0.26c	12.69 ± 0.13c	11.89 ± 0.15c	0.0867 ± 0.0035c
8	8.24 ± 0.45bc	15.94 ± 0.22bc	3.87 ± 0.03b	8.11 ± 0.20b	8.33 ± 0.23b	13.49 ± 0.12b	12.22 ± 0.14b	0.0918 ± 0.0031b
12	8.64 ± 0.42ab	16.66 ± 0.20ab	4.19 ± 0.03a	8.64 ± 0.18a	8.87 ± 0.21a	13.90 ± 0.11a	12.78 ± 0.13a	0.0968 ± 0.0029a
16	8.86 ± 0.40a	17.71 ± 0.18a	4.56 ± 0.02a	8.96 ± 0.17a	9.26 ± 0.20a	14.66 ± 0.10a	13.56 ± 0.12a	0.0989 ± 0.0027a
20	8.78 ± 0.43a	17.22 ± 0.19ab	4.56 ± 0.03a	8.13 ± 0.21b	8.79 ± 0.22ab	14.21 ± 0.11ab	13.18 ± 0.13ab	0.0940 ± 0.0030ab
LSD %5	2.33	0.60	0.07	0.54	0.62	0.30	0.35	0.0081

Values within a column (Mean ± SD) followed by different lowercase letters are significantly different according to LSD test at *p* ≤ 0.05.

Where: Vitamin (C) as L-Ascorbic acid (mg 100 g^−1^ FW), Vitamin (A) as Beta carotene g 100 g^−1^ FW

Table 7 details the impact of various cobalt treatments on the percentage composition of essential oils in sweet fennel bulbs 120 days after sowing in field experiments during the second season. The total identified compounds under control conditions represented 74.81% of the total essential oil composition. This is consistent with previous essential oil analyses, where the total percentage of identified compounds May fall short of 100% due to analytical limitations such as low-abundance constituents, co-eluting peaks, or the absence of reference spectra in existing databases. While the major compounds were clearly identified and quantified, trace or unidentified compounds likely contributed to the remaining percentage. Future studies employing more sensitive detectors or complementary analytical platforms (e.g., GC×GC-MS or HRMS) May help provide a more comprehensive characterization of the essential oil profile. The most significant improvements in essential oil composition were observed at a Cobalt concentration of 16 Mg L^−1^. For *α*-pinene, the highest content was 3.59%, representing an increase of approximately 9.1% compared to the control (3.29%). Camphene peaked at 0.94%, showing a 40.3% rise relative to the control (0.67%). *β*-Pinene reached its maximum at 1.52%, an 18.8% increase compared to the control (1.28%). Myrcene content was highest at 1.71%, reflecting a 19.6% increase compared to the control (1.43%). *α*-Phellandrene content peaked at 1.53%, a 19.5% rise relative to the control (1.28%). Limonene reached its maximum at 3.15%, an 8.2% increase compared to the control (2.91%). *γ-*Terpinene content was highest at 1.17%, showing a 23.2% increase compared to the control (0.95%). p-Cymene peaked at 2.13%, a 12.7% rise relative to the control (1.89%). Fenchone content reached its maximum at 7.19%, and 4.5% increase compared to the control (6.88%). Estragole peaked at 5.32%, showing a 7.0% rise relative to the control (4.97%). Cis-anethole content was highest at 0.75%, a 44.2% increase compared to the control (0.52%). Trans-Anethole content showed a slight increase, peaking at 47.74% at 12 Mg L^−1^, but then slightly decreased to 47.51% at 16 Mg L^−1^, still close to the control (47.51%). Anisalketone content reached its maximum at 1.96% at 20 Mg L^−1^, a significant increase compared to the control (1.23%). These findings demonstrate that a Cobalt treatment of 16 Mg L^−1^ significantly enhances the composition of essential oils in sweet fennel bulbs across various metrics. However, it is noteworthy that at higher concentrations (20 Mg L^−1^), some essential oil components showed a slight decline, indicating a potential toxicity threshold. For example, *α*-pinene decreased to 3.55%, and Trans-Anethole content dropped to 47.50%, suggesting that excessive Cobalt levels May adversely affect the essential oil composition of sweet fennel bulbs. anisaldehyde was present only in trace amounts (denoted as “T” in the table) across all Cobalt treatments, including the control. This means that the concentration of anisaldehyde was less than 0.1% in all cases. There was no significant change in anisaldehyde content with increasing Cobalt concentrations, as it remained at trace levels throughout the experiment. This indicates that Cobalt treatment did not have a measurable impact on the anisaldehyde content in the essential oils of sweet fennel bulbs.

**Table 7:** Percentage composition of the essential oils of sweet fennel bulb which planted in field experiments as affected by cobalt addition after 120 days from sowing (in the second season)

Essential oil (%)	Cobalt treatments (mg L^−1^)					
	Control	4	8	12	16	20
Cis-anethole	0. 52	0.56	0.62	0.68	0.75	0.75
Camphene	0.67	0.73	0.79	0.86	0.94	0.94
*γ-*Terpinene	0.95	0.98	1.05	1.09	1.17	1.15
*α*-Pinene	3.29	3.34	3.41	3.59	3.59	3.55
*α*-Phellandrene	1.28	1.33	1.40	1.46	1.53	1.51
*β*-Pinene	1.28	1.32	1.38	1.44	1.52	1.49
Anisalketone	1.23	1.28	1.35	1.43	1.49	1.96
Myrcene	1.43	1.47	1.53	1.61	1.71	1.68
Limonene	2.91	2.94	2.99	3.06	3.15	3.15
p-Cymene	1.89	1.92	1.97	2.04	2.13	2.10
Fenchone	6.88	6.93	6.99	7.07	7.19	7.18
Estragole	4.97	5.06	5.14	5.23	5.32	5.50
Trans-Anethole	47.51	47.56	47.64	47.74	47.51	47.50
Anisaldehyde	T	T	T	T	T	T

Where: T: Traces (<0.1%)

**Fig. 1:**
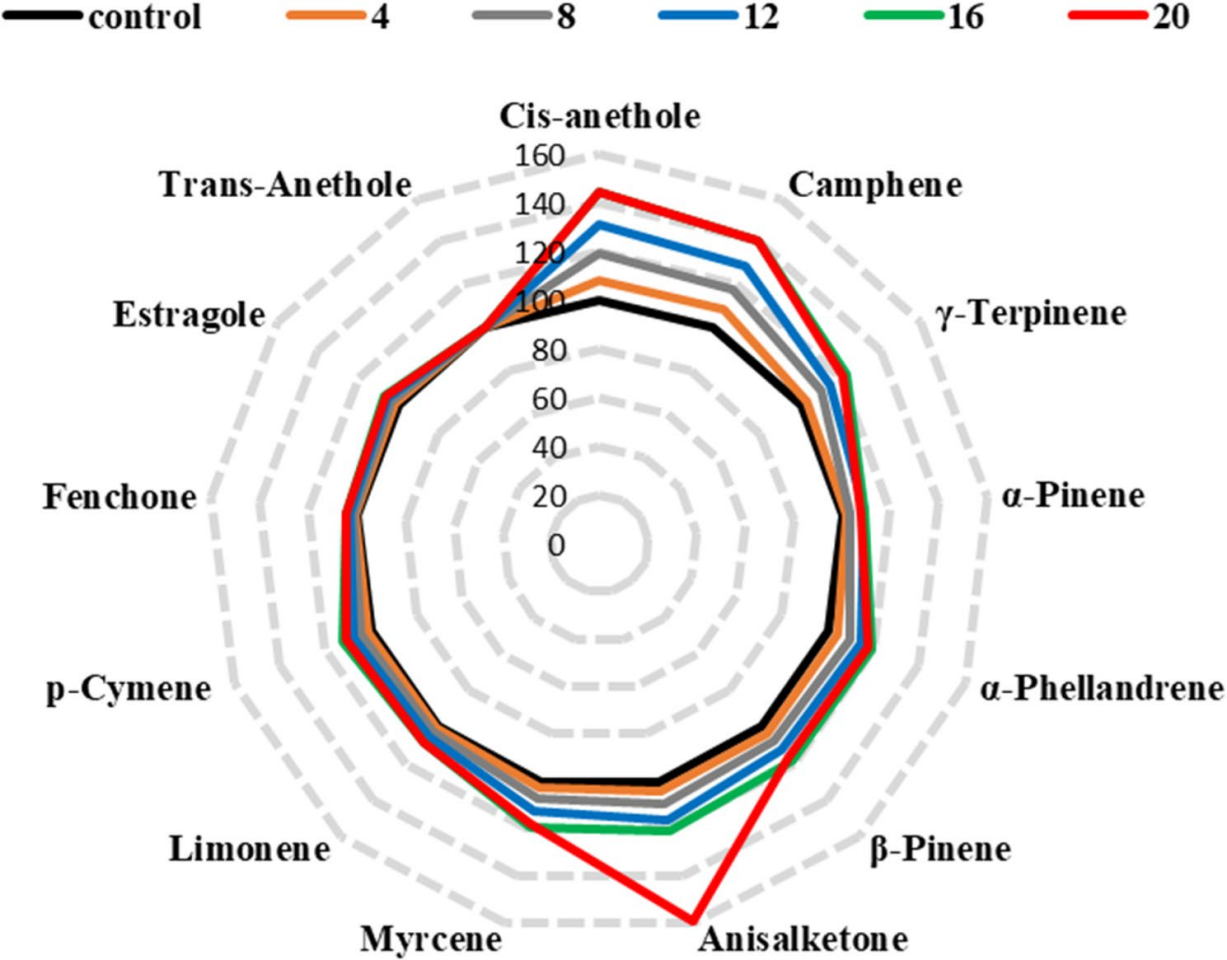
A radar diagram illustrating relative deviations from the control (a black circle of the relative value of 100 %) of all essential oils in sweet fennel bulbs 120 days after sowing in field experiments during the second season

In the current study, plants treated with cobalt at concentrations of 16 and 20 mg L^−1^ showed higher levels of most essential oils compared to the other treatments (Fig. 1), while the essential oils (*γ-*Terpinene, *α*-Pinene, *β*-Pinene, Myrcene, p-Cymene, Fenchone, Estragole, and Trans-Anethole) had their highest values ​​at concentrations of 16 mg L^−1^ and then decreased at concentrations of 20 mg L^−1^.

## Discussion

The results of this study demonstrate that cobalt (Co) application at an optimal concentration of 16 mg L⁻¹ significantly enhances the vegetative growth, yield, and chemical composition of sweet fennel (*Foeniculum vulgare*) plants. However, higher concentrations (18–20 mg L⁻¹) led to a decline in some growth and yield parameters, suggesting a potential toxicity threshold. These findings align with recent studies that highlight the dual role of cobalt as both a beneficial micronutrient and a potential toxin at elevated levels.

In both pot and field experiments, cobalt treatment at 16 mg L⁻¹ resulted in substantial improvements in vegetative growth parameters, including plant height, leaf number, and fresh and dry weights of leaves and bulbs. The increase in plant height (54.03% in pots and 44.70% in field experiments) and leaf number (100.66% in pots and 107.56% in field experiments) indicates that cobalt plays a critical role in promoting cell division and elongation, likely through its involvement in enzymatic activities and hormonal regulation [30]. Similar findings to ours indicate that optimal cobalt levels significantly enhanced growth by improving photosynthetic efficiency and nutrient uptake in broad bean [31], broccoli [32], and summer squash [33]. Similarly, Gad and Hassan [34] confirmed that cobalt had a significant positive impact on the growth and yield parameters of sweet pepper, with treated plants outperforming non-treated ones. The significant increase in fresh and dry weights further supports the role of cobalt in enhancing biomass accumulation, as noted by Inayat, Mehmood [35] in their study on cobalt’s effects on radish growth. However, the decline in growth metrics at higher concentrations (18–20 mg L⁻¹) suggests that excessive cobalt may disrupt cellular processes, leading to oxidative stress, nutrient deficiencies, and impairment of the photosynthetic apparatus [36, 37]. These results align with the findings of Gad [38], who reported that cobalt positively influenced the synthesis of key endogenous hormones in tomato plants, such as auxins and gibberellins, significantly enhancing growth. Additionally, cobalt reduced the activity of catalase and peroxidase enzymes, which are known to stimulate plant respiration, thereby contributing to improved plant performance [39].

The yield parameters, including bulb height, diameter, weight, and total yield per feddan, were also maximized at 16 mg L⁻¹ cobalt. The increase in bulb weight (46.5%) and total yield (20.2%) highlights the positive impact of cobalt on nutrient partitioning and storage organ development. The cobalt application improved yield attributes of some plants by enhancing nitrogen metabolism and assimilate translocation [40]. The observed decline in yield metrics at 20 mg L⁻¹ cobalt further reinforces the concept of a toxicity threshold, beyond which cobalt may interfere with nutrient uptake and metabolic processes, as noted by Ulhassan, Shah (41) in their study on cobalt’s effects on crop productivity. Cobalt application at 10 mg kg⁻¹ significantly improved growth parameters, bulb yield, length, and quality—including nutrient and essential oil content—in two onion cultivars. Both bulb diameter and weight were markedly higher compared to the control [14]. However, concentrations exceeding 10 mg kg⁻¹ substantially diminished these beneficial effects.

Cobalt treatment at 16 mg L⁻¹ significantly enhanced the macronutrient (N, P, K) and micronutrient (Mn, Zn, Cu) content of fennel bulbs. These results are consistent with the findings of Jayakumar, Jaleel (11), who reported that cobalt at 50 mg kg⁻¹ soil enhanced the N, P, and K content in black gram seeds compared to the control. The increase in nitrogen content (62.4%) is particularly noteworthy, as cobalt is known to facilitate nitrogen metabolism and assimilation, as reported by Hu, Wei (8) and Gericó, Tavanti [41]. The improvement in phosphorus and potassium uptake further underscores the role of cobalt in enhancing nutrient availability and utilization, as highlighted by Elshamly and Nassar [42]. However, the decline in iron content at higher cobalt concentrations suggests a possible antagonistic interaction between cobalt and iron, which could disrupt iron-dependent enzymatic activities and lead to nutrient imbalances, as some researchers reported [43–45].

The cobalt content in fennel bulbs treated with 16 mg L⁻¹ reached 8.06 mg kg⁻¹, which is within safe limits for human consumption. According to the World Health Organization (WHO) and Food and Agriculture Organization (FAO), the tolerable daily intake (TDI) for cobalt is 0.05 µg kg^−1^ body weight per day, with typical dietary exposure from crops being much lower [46, 47]. However, prolonged consumption of cobalt-rich crops should be monitored, as excessive intake may lead to thyroid dysfunction or cardiomyopathy in sensitive individuals [46, 48]. Our findings suggest that 16 mg L⁻¹ cobalt application optimizes fennel growth and quality without exceeding safety thresholds, but further long-term bioavailability studies are recommended to confirm minimal health risks.

Biochemical analysis revealed that 16 mg L⁻¹ cobalt treatment significantly enhanced total proteins (62.3%), carbohydrates (25.5%), soluble sugars, phenolic compounds (34.6%), vitamins (C: 18.5%; A: 19.9%), and oil content, indicating stimulated metabolic activity [48], improved antioxidant capacity [49], and increased synthesis of health-promoting compounds [50]. However, higher cobalt concentrations reversed these benefits, suggesting metabolic impairment and reduced nutritional quality [8], potentially due to cobalt’s inhibition of chlorophyll biosynthesis enzymes (5-aminolevulinic acid and protoporphyrin pathways) and consequent photosynthetic decline [51].

The essential oil composition of fennel bulbs was also positively influenced by cobalt treatment at 16 mg L⁻¹. Significant increases were observed in key components such as *α*-pinene, camphene, *β*-pinene, and fenchone, which contribute to the aroma and medicinal properties of fennel. Similar findings were reported by Khalid and Ahmed [52], who demonstrated that cobalt application enhanced the biosynthesis of essential oils in *Nigella sativa* L. plants. The increase in trans-anethole, a major bioactive compound in fennel, further underscores the potential of cobalt to enhance the commercial value of the plants. These findings are consistent with previous studies on the effects of cobalt on essential oil composition. Gad, Abd El-Moez [53] observed that all cobalt treatments significantly enhanced the essential oil content of rosemary (*Rosmarinus officinalis* L.) compared to the control. Similarly, Aziz, Gad (55) reported that cobalt increased essential oil yield in sweet basil (*Ocimum basilicum* L.), with the highest values obtained at 16 ppm. An earlier study by the same authors [55], also demonstrated a significant increase in essential oil yield in peppermint (*Mentha piperita* L.) following cobalt application.

The enhancement of essential oil constituents such as *α*-pinene, Limonene, and trans-anethole at 16 mg L⁻¹ cobalt can be attributed to cobalt’s role in modulating secondary metabolism, particularly through its influence on enzymatic activity within terpenoid and phenylpropanoid biosynthetic pathways [54]. Cobalt acts as a cofactor for several metalloenzymes and may stimulate key enzymes such as phenylalanine ammonia-lyase (PAL) [56], which initiates the phenylpropanoid pathway, and geranyl pyrophosphate synthase (GPPS), which is involved in monoterpene biosynthesis [8]. This activation likely enhances the synthesis of volatile compounds derived from these pathways, including trans-anethole and *α*-pinene [57]. Additionally, at higher concentrations (e.g., 20 mg L⁻¹), the observed decline in several volatile constituents may reflect cobalt-induced toxicity, which can disrupt cellular homeostasis, impair enzyme function, and suppress secondary metabolic flux. Excess cobalt may generate elevated levels of reactive oxygen species (ROS) beyond the threshold of beneficial signaling, leading to oxidative damage and inhibition of biosynthetic pathways [58]. These findings suggest a dose-dependent dual role of cobalt—acting as a stimulant at moderate levels and as a stressor or inhibitor at higher doses—highlighting the importance of optimizing micronutrient application for maximizing secondary metabolite output while minimizing potential toxicity.

The observed increase in estragole concentration in sweet fennel essential oil under cobalt supplementation is indeed noteworthy, particularly given estragole’s classification as a compound with potential carcinogenicity. This elevation may be explained by cobalt’s role in modulating plant secondary metabolism, particularly under mild stress conditions [59]. Cobalt is known to influence the production of reactive oxygen species (ROS) and can act as a signaling molecule that triggers the biosynthetic pathways of secondary metabolites, including phenylpropanoids such as estragole [60, 61]. It is plausible that cobalt-induced stress responses upregulate enzymes involved in the shikimate and phenylpropanoid pathways, ultimately enhancing estragole biosynthesis [36]. Therefore, while cobalt application may improve essential oil yield and composition, it can also inadvertently increase the levels of certain constituents like estragole.

These findings underscore the importance of balancing productivity enhancements with safety considerations, particularly for crops intended for food and pharmaceutical use. The results of this study indicate that foliar application of cobalt at 16 mg L⁻¹ may serve as an effective agronomic strategy to improve growth performance, yield, and nutritional quality in sweet fennel. However, to ensure the broader applicability of these outcomes, further research is necessary to validate the results under diverse environmental and agronomic conditions. Moreover, a deeper investigation into the physiological and molecular mechanisms underlying cobalt’s effects is warranted. Importantly, the potential for cobalt accumulation in edible plant tissues must be thoroughly assessed to safeguard consumer health and ensure regulatory compliance.

## Conclusions

The study demonstrates that cobalt supplementation at a concentration of 16 mg L⁻¹ significantly enhances the vegetative growth, yield, and chemical composition of sweet fennel. This optimal treatment resulted in substantial improvements in plant height, leaf number, fresh and dry weights, bulb dimensions, and total yield. Additionally, the nutritional quality of fennel bulbs was enhanced, with increased levels of macronutrients, micronutrients, total proteins, carbohydrates, soluble sugars, phenolic content, vitamins C and A, and oil content. The composition of essential oils also improved, with notable increases in key components such as *α*-pinene, camphene, and fenchone. However, higher cobalt concentrations (20 mg L⁻¹) led to a slight decline in some growth and chemical metrics, indicating a potential toxicity threshold. These findings suggest that while cobalt can be a beneficial micronutrient for sweet fennel cultivation, careful management of application rates is essential to avoid adverse effects. The results provide valuable insights for optimizing cobalt use in agricultural practices to enhance crop productivity and quality.

## Supplementary Information

Supplementary Material 1.

## Data Availability

Data is provided within the manuscript and supplementary data.
