# Supplementary material for: Optimizing sweet fennel growth and quality: the impact of cobalt supplement on vegetative growth, yield, and chemical composition
Source: BMC Plant Biol. 2025 Sep 16;25:1205. doi: 10.1186/s12870-025-07314-y (PMC12439422; doi:10.1186/s12870-025-07314-y)
Supplement: Supplementary file 1 — Supplementary Material 1. [file 12870_2025_7314_MOESM1_ESM.pdf]

Table S1. Bi-weekly climatic averages during the 2022/2023 growing season at El-Nubaryia, Egypt: Temperature (°C), relative humidity (%), and rainfall (mm).

**Period**	**Avg. Max Temp**	**Avg. Min Temp**	**Relative Humidity (%)**	**Rainfall (mm)**	**Notes**
**Aug 1–15**	35–37°C	24–26°C	60–70% (AM), 40–50% (PM)	**0 mm**	Peak summer heat
**Aug 16–31**	34–36°C	23–25°C	65–75% (AM), 45–55% (PM)	**0–1 mm**	Slight humidity rise
**Sep 1–15**	33–35°C	22–24°C	65–80% (AM), 45–60% (PM)	**0 mm**	Early autumn transition
**Sep 16–30**	32–34°C	21–23°C	70–85% (AM), 50–65% (PM)	**0–1 mm**	Dew common at dawn
**Oct 1–15**	30–32°C	19–21°C	70–85% (AM), 50–60% (PM)	**0–2 mm**	Cooling begins
**Oct 16–31**	28–30°C	17–19°C	75–88% (AM), 55–65% (PM)	**1–3 mm**	First light rains
**Nov 1–15**	26–28°C	15–17°C	75–90% (AM), 55–70% (PM)	**3–6 mm**	Rainfall increases
**Nov 16–30**	24–26°C	13–15°C	80–92% (AM), 60–75% (PM)	**5–10 mm**	Foggy mornings
**Dec 1–15**	21–23°C	11–13°C	80–90% (AM), 60–70% (PM)	**8–15 mm**	Winter onset
**Dec 16–31**	19–21°C	9–11°C	85–92% (AM), 65–75% (PM)	**10–20 mm**	Highest humidity
**Jan 1–15**	18–20°C	8–10°C	85–93% (AM), 65–75% (PM)	**12–20 mm**	Coldest nights
**Jan 16–31**	17–19°C	7–9°C	80–90% (AM), 60–70% (PM)	**10–18 mm**	Moderate rain

Source: Egyptian Meteorological Authority (EMA) and National Research Centre (NRC) Agrometeorology Unit

Table S2. Bi-weekly climatic averages during the 2023/2024 growing season at El-Nubaryia, Egypt: Temperature (°C), relative humidity (%), and rainfall (mm).

Period	Avg. Max Temp	Avg. Min Temp	Relative Humidity (%)	Rainfall (mm)	Notes
Aug 1–15	34–36°C	23–25°C	60–75% (AM), 40–55% (PM)	0 mm	Dry & hot
Aug 16–31	33–35°C	22–24°C	65–80% (AM), 45–60% (PM)	0–1 mm	Slight cooling
Sep 1–15	32–34°C	21–23°C	70–85% (AM), 50–65% (PM)	0 mm	Humidity rises
Sep 16–30	31–33°C	20–22°C	75–88% (AM), 55–70% (PM)	0–1 mm	Morning dew
Oct 1–15	29–31°C	18–20°C	75–90% (AM), 55–65% (PM)	1–3 mm	First drizzle
Oct 16–31	27–29°C	16–18°C	80–92% (AM), 60–70% (PM)	2–5 mm	Rain begins
Nov 1–15	25–27°C	14–16°C	80–93% (AM), 60–75% (PM)	4–8 mm	Foggy conditions
Nov 16–30	23–25°C	12–14°C	85–95% (AM), 65–80% (PM)	6–12 mm	Wet spells
Dec 1–15	20–22°C	10–12°C	85–95% (AM), 70–80% (PM)	10–18 mm	Peak winter rains
Dec 16–31	18–20°C	8–10°C	85–95% (AM), 65–75% (PM)	12–22 mm	Chilly nights
Jan 1–15	17–19°C	7–9°C	80–90% (AM), 60–70% (PM)	10–20 mm	Moderate cold
Jan 16–31	16–18°C	6–8°C	80–90% (AM), 60–70% (PM)	8–15 mm	Rainfall ta

Source: Egyptian Meteorological Authority (EMA) and National Research Centre (NRC) Agrometeorology Unit
